# Korean Red Ginseng slows coreceptor switch in HIV-1 infected patients

**DOI:** 10.1016/j.jgr.2022.06.003

**Published:** 2022-07-06

**Authors:** Young-Keol Cho, Jung-Eun Kim, Jinny Lee

**Affiliations:** Departments of Microbiology, University of Ulsan College of Medicine, Asan Medical Center, Seoul, Republic of Korea

**Keywords:** coreceptor tropism switch, HIV-1 *env* gene, False positive rate, Korean Red Ginseng

## Abstract

**Background:**

Human immunodeficiency virus-1 (HIV-1) that binds to the coreceptor CCR5 (R5 viruses) can evolve into viruses that bind to the coreceptor CXCR4 (X4 viruses), with high viral replication rates governing this coreceptor switch. Korean Red Ginseng (KRG) treatment of HIV-1 infected patients has been found to slow the depletion of CD4+ T cells. This study assessed whether the KRG-associated slow depletion of CD4+ T cells was associated with coreceptor switching.

**Methods:**

This study included 146 HIV-1-infected patients naïve to antiretroviral therapy (ART) and seven patients receiving ART. A total of 540 blood samples were obtained from these patients over 122 ± 129 months. Their *env* genes were amplified by nested PCR or RT-PCR and subjected to direct sequencing. Tropism was determined with a 10% false positive rate (FPR) cutoff.

**Results:**

Of the 146 patients naïve to ART, 102 were KRG-naïve, and 44 had been treated with KRG. Evaluation of initial samples showed that coreceptor switch had occurred in 19 patients, later occurring in 38 additional patients. There was a significant correlation between the amount of KRG and FPR. Based on initial samples, the R5 maintenance period was extended 2.35-fold, with the coreceptor switch being delayed 2.42-fold in KRG-treated compared with KRG-naïve patients. The coreceptor switch occurred in 85% of a homogeneous cohort. The proportion of patients who maintained R5 for ≥10 years was significantly higher in long-term slow progressors than in typical progressors.

**Conclusion:**

KRG therapy extends R5 maintenance period by increasing FPR, thereby slowing the coreceptor switch.

## Graphical abstract

**Figure:**
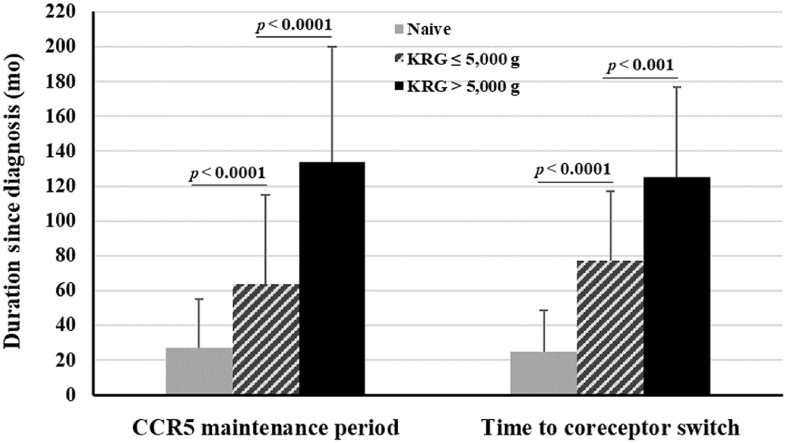

## 1 Introduction

Human immunodeficiency virus 1 (HIV-1) uses two receptors to enter cells, with the main receptor being CD4 and the coreceptors being CCR5 (R5) and CXCR4 (X4) [1]. Viruses using R5 (R5 viruses) are generally present over the entire course of infection, whereas those using X4 (R5X4 or X4 viruses) emerge in approximately 50–70% of infected individuals during later stages of infection [2,3]. X4 viruses have been shown to evolve from pre-existing R5 viral populations, with coreceptor switching being associated with high replication rates, but not selective pressure [3]. This coreceptor switching is more frequent in patients who have failed antiretroviral therapy (ART) [4], with immune activation correlating with X4 tropism [5]. A simulation study of a stochastic model showed that coreceptor switching occurs from 6 to more than 27 years after infection [6]. The emergence of HIV-1 populations that switch coreceptors from R5 to X4 has been associated with the depletion of CD4+ T cells and disease progression toward acquired immune deficiency syndrome (AIDS). HIV-1 coreceptor usage is therefore of central pathological and clinical significance [7].

False positive rate (FPR) has been defined as the probability of erroneously classifying an R5-virus as an X4-virus. A low FPR at baseline, as determined by the Geno2pheno_[coreceptor]_ algorithm, has been shown to predict the evolution of tropism from the R5 to the X4 coreceptor [8], but no other correlates of tropism switch have yet been identified [8].

Treatment of HIV-1-infected patients with Korean Red Ginseng (KRG) has been shown to slow the depletion of CD4+ T cells [9], resulting in the maintenance of sufficient CD4+ T cell counts for more than 20 years in the absence of ART [10]. Thus, KRG prolonged the survival of HIV-1-infected patients [11] by reducing hyperimmune activation [12] and induction of genetic defects [[13], [14], [15], [16], [17], [18]]. It was unclear, however, whether KRG treatment affects the coreceptor tropism switch. The present study therefore evaluated whether treatment of HIV-1 infected patients with KRG could affect the switching of coreceptors from R5 to X4.

## 2 Method and materials

### 2.1 Ethics statement

This study, which was conducted in accordance with the Declaration of Helsinki, was approved by the Institutional Review Board of Asan Medical Center of the University of Ulsan College of Medicine, Seoul, Korea (Code 2012-0390). All subjects provided written informed consent before inclusion in this study.

### 2.2 Patients

The study cohort included 146 patients who had been diagnosed with HIV-1 infection and were naïve to ART, and seven patients on ART, including three patients with hemophilia (patients 15, 19, and 20). Thirty-nine patients had been treated with KRG since December 1991, making a significant proportion long-term slow progressors (LTSP) [[11], [12], [13]]. Of the patients in this study, 127 and 24 were infected with B and non-B subtypes, respectively, of HIV-1, including four D and seven CRF01-AE, respectively. Of the patients infected with subtype B, 20 were hemophiliacs (HP) who had been infected with a single source of KSB HIV-1 via domestic clotting factor (DCF) 9 between 1990 and 1994 [[19], [20], [21]] (Fig. S1).

### 2.3 CD4+ and CD8+ T cell counting

PBMCs were stained with phycoerythrin- and fluorescein isothiocyanate (FITC)-conjugated antibodies to CD4 and CD8 antigens, respectively (Simultest reagents, Becton-Dickenson [BD], CA, USA) [9], and subjected to flow cytometry using a FACScan flow cytometer (BD).

### 2.4 KRG treatment

The outpatient-based KRG trial in HIV-1-infected patients was begun at the Korean NIH in late 1991, as described [9]. The KRG used in this study was a commercial product prepared from 6-year-old KRG roots by the Korea Ginseng Corporation. Beginning in 1991, patients were instructed to take six capsules, each containing 300 mg KRG, three times per day, for a total daily dose of 5.4 g; beginning in 2011, patients were instructed to take four, each containing 500 mg KRG, three times per day, for a total daily dose of 6.0 g. The amounts of KRG are listed in Table 1, Table 2.

**Table 1:** Baseline Characteristics in the KRG-naive and KRG-treated Groups Before ART

Patient characteristics	Naïve (%)	KRG (%)	P-value
No. of patients	102	44	
Age at diagnosis	28 ± 10	32 ± 7	**<0.05**
Male:female	87(85.3):15(14.7)	39(88.6):5(11.4)	
Follow up period since diagnosis	18 ± 20	42 ± 27	**<0.0001**
Year of diagnosis			
1987-1993	87(85.3)	41(93.2)	
1994-2003	15(14.7)	3(6.8)	
Transmission route			**>** 0.05
Homosexual contact	47(46.1)	27(61.4)	
Heterosexual contact	36(35.3)	15(34.1)	
Blood transfusion or product	19(18.6)	2(4.5)	
Amount of KRG supplied (g)	0	2,025 ± 3,244	
CD4^+^ T cell count at tropism	449 ± 236	380 ± 238	> 0.05
> 200/μL	87(85.3)	37(84.1)	
< 200/μL	15(14.7)	7(15.9)	
FPR at the first sample			> 0.05
X4 ≤ 10 %	14(13.7)	5(11.4)	
R5 > 10 %	88(86.3)	39(88.6)	
No. of zidovudine treatment	10(9.8)	18(40.9)	**<0.0001**
Unknown	14(13.7)	1(2.3)	
Virus subtype			> 0.05
B	88(86.3)	34(77.3)	
Non-B	14(13.7)	10(22.7)	

KRG, Korean Red Ginseng.

**Table 2:** CCR5 Maintenance period and time to coreceptor switch are prolonged by KRG therapy

Item	Naïve (n = 58)		KRG (n = 88)	P-value
CCR5 maintenance period (mo)	27 ± 28	80 ± 67 (4,397 ± 5,964 g)		< 0.0001
Time to coreceptor switch (mo)	25 ± 23	87 ± 44 (3,510 ± 3,542 g)		< 0.0001

KRG, Korean Red Ginseng.

### 2.5 Measurement of viral load

HIV-1 RNA copy numbers in serum (before 1997) were measured using AMPLICOR HIV-1 monitoring kits (Roche Diagnostics Systems, Branchburg, NJ, USA). Copy numbers in sera were converted to plasma equivalent numbers per ml [9].

### 2.6 RNA/DNA preparation and *env* gene amplification

Total RNA was extracted from 300 μl serum samples using QIAamp UltraSens Viral RNA kits (Qiagen, Hilden, Germany). RNA was reverse-transcribed using Superscript III reverse transcriptase (Invitrogen, Carlsbad, CA, USA). DNA was extracted from 400 μl PBMC samples using a QIAamp DNA Mini kit (Qiagen), and 5 μl aliquots of DNA were used for nested PCR [21,22]. The *env* gene was amplified via nested PCR with TaKaRa LA-Taq (Takara Bio Inc., Shiga, Japan). The first and second PCR reactions were performed in reaction mixtures of 25 μl and 50 μl, respectively. The outer primer pairs were OWE1 and OWE2, whereas the inner primer pairs were OWE3 and OWE4 [21,22]. The amplicons were directly sequenced using an Applied Biosystems 3730XL Analyzer (Foster City, CA, USA).

### 2.7 Envelope V3 loop sequencing and coreceptor tropism determination

Proviral DNA was used to amplify and sequence the HIV-1 envelope C2V3 region or full-length *env* gene as previously described [[21], [22], [23]]. HIV-1 coreceptor tropism was determined from the V3 loop sequences using the Geno2pheno_[coreceptor]_ (G2P) algorithm (http://coreceptor.geno2pheno.org/index.php). G2P was set at FPR of 10%, with patients having an FPR ≤10% considered infected with X4-tropic viruses [24], based on European guidelines [25]. In this study, prediction was based on clonal training data and V3 sequences alone.

### 2.8 Statistical analysis

Data were expressed as means ± standard deviation (SD) and compared by Student's two-tailed paired t-tests, Chi-square tests, Fisher's exact tests, and Spearman's correlation coefficient (*r*) tests, as appropriate. All statistical analyses were performed using MedCalc software (Ostend, Belgium), with statistical significance defined as *p* < 0.05.

### 2.9 Sequences data

The GenBank accession numbers for the sequences identified in this study are AY581320-423, KU869531-620, KU896057-129, KX960961-980, AJ417408-31, and ON567771-ON568120.

## 3 Results

HIV-1 coreceptor tropism was determined in serum or proviral DNA samples obtained from 146 patients naïve to ART and from seven patients on ART. In addition, 46 patients were followed-up while receiving ART for 150 ± 80 months. The total number of samples was 540 [402 before and 138 during ART or ginseng-based combination therapy (GCT)] over a mean of 122 ± 129 months (median; 60 months). The *env* gene was sequenced in the patients naïve to ART [[21], [22], [23]]. Of the 146 patients naïve to ART, 102 were naïve to KRG and 44 had been treated with KRG (Table 1).

The demographic characteristics at the time of obtaining the first sample were compared in the 102 patients naïve to KRG and the 44 who had been treated with KRG. Age at diagnosis, follow-up period following diagnosis, and the number of zidovudine treatments were significantly greater in the KRG-treated than in the KRG-naïve group (Table 1). The proportion of patients with FPR ≤10% was similar in the two groups. In addition to 24 patients who were positive for coreceptor switching in the first sample, 38 showed subsequent coreceptor switching. Taken together, a total of 62 (40.5%) of the 153 patients were positive for X4 or R5X4 (coreceptor switching).

### 3.1 Changes in FPR before and after KRG treatment

FPR gradually and significantly decreased over time, although the patients were followed-up for a mean of of approximately 6 years [7]. FPR in 19 of 28 patients was higher after short-term KRG treatment (3 years or less) than before KRG. In addition, FPR gradually and significantly increased over long time in several KRG-treated patients.

Because an increase in the number of samples in which tropism is determined has been associated with a more accurate analysis of the effect on coreceptor switch, the effects of KRG dosage was analyzed by dividing patients with tropism determined in one, two or more, and three or more samples. Dosages of KRG in these three groups were 2,025 ± 3,244 g, 4,632 ± 5,168 g, and 5,248 ± 5,516 g, respectively, with dosages being significantly higher in the second and third groups than in the first group (P < 0.01). The times from diagnosis to sample acquisition in these three groups were 42 ± 27, 92 ± 58, and 96 ± 58 months, respectively.

**Fig. 1:**
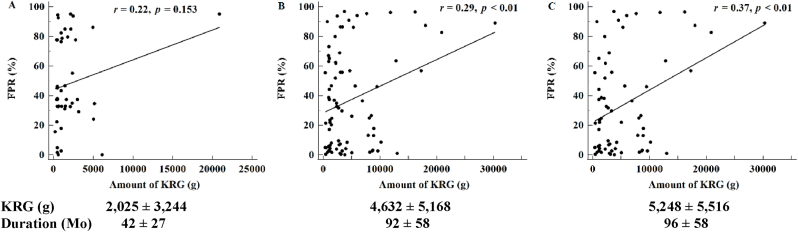
Correlation between false positive rate (FPR) and the total dosage of Korean Red Ginseng (KRG). (A) Lack of correlation in the 44 patients with tropism evaluated in the initial sample. (B, C) Significant correlations in (B) the 77 patients with tropism determined in two or more samples and in (C) the 58 patients with tropism determined in three or more samples. The dosages of KRG were significantly higher in patients with tropism determined in two or more (4,632 ± 5,168 g) and three or more (5,248 ± 5,516 g) samples than in the initial sample (2,025 ± 3,244 g) (P < 0.01).

Analyses of initial samples showed a trend in correlation between FPR and amount of KRG (*r* = 0.22, P = 0.153) (Fig. 1A). Correlations were significant, however, in the 77 patients with tropism assessed in two or more samples (*r* = 0.29, P < 0.01; Fig. 1B), and in the 58 patients with tropism assessed in three or more samples (*r* = 0.37, P < 0.01; Fig. 1C). Taken together, these findings indicate that higher KRG doses were associated with more significant increases in FPR.

### 3.2 Effect of KRG treatment on R5 maintenance period

**Fig. 2:**
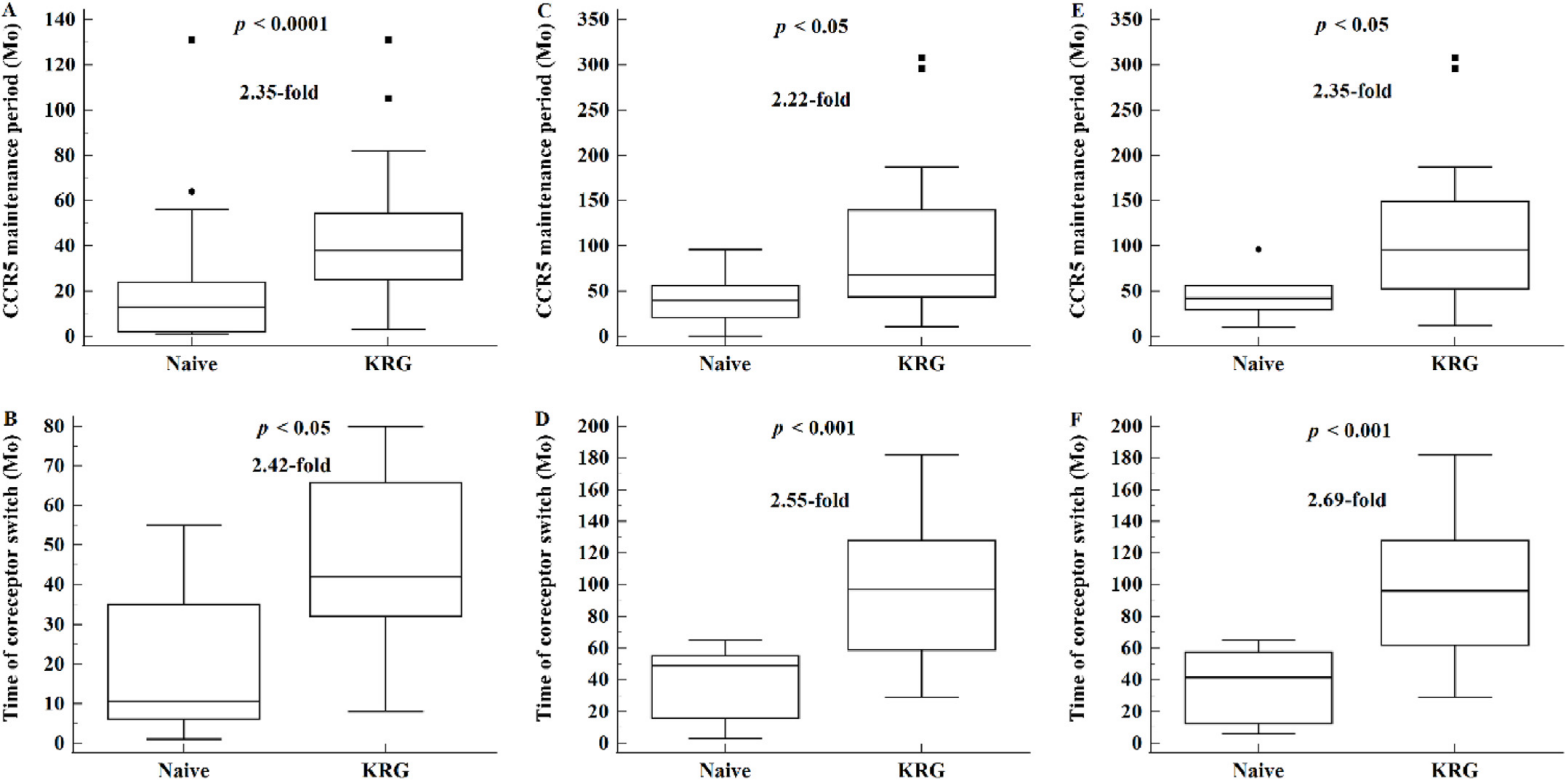
Comparisons of (A, C, E) CCR5 maintenance period and (B,D,F) time to coreceptor switch in KRG-treated and KRG-naïve patients, as determined in (A,B) the initial samples of all 146 patients before antiretroviral therapy (ART), (C,D) the 77 patients with tropism determined in two or more samples, and (E,F) the 58 patients with tropism determined in three or more samples. In all three groups, the CCR5 maintenance period and time of coreceptor switch were significantly longer in KRG-treated than in KRG-naïve patients (P < 0.05 each).

KRG treatment has been shown to slow the depletion of CD4+ T cells [[9], [10], [11]], suggesting that KRG treatment may affect the timing of tropism switching. Analyses of initial samples in 146 patients showed an association between KRG treatment and coreceptor switching, with 127 showing R5 alone and 19 showing coreceptor switching prior to ART (Table 1). The R5 maintenance period was significantly longer (2.35-fold) in KRG-treated than in KRG-naïve patients (42 ± 27 versus 18 ± 20 months; P < 0.0001) (Fig. 2A), with the time of coreceptor switching also being significantly delayed (2.42-fold) by 19 ± 19 months in KRG-naïve patients (P < 0.01) (Fig. 2B). Similar results were observed in the 77 patients with tropism determined in two or more samples and in the 58 patients with tropism determined in three or more samples. Relative to KRG-naïve patients in these two groups, coreceptor switch was significantly delayed by 2.22- and 2.35-fold, respectively, in KRG-treated patients (P < 0.05 for both). The times from diagnosis to coreceptor switch in patients with tropism determined in one, two or more, and three or more samples were 45 ± 23, 103 ± 40, and 102 ± 41 months, respectively, and the dosages of KRG were 1,601 ± 2,224 g, 3,899 ± 3,398 g, and 4,101 ± 3,446 g, respectively. Taken together, these findings show that KRG treatment significantly extended the R5 maintenance period and, consequently, signficantly slowed coreceptor switch.

**Fig. 3:**
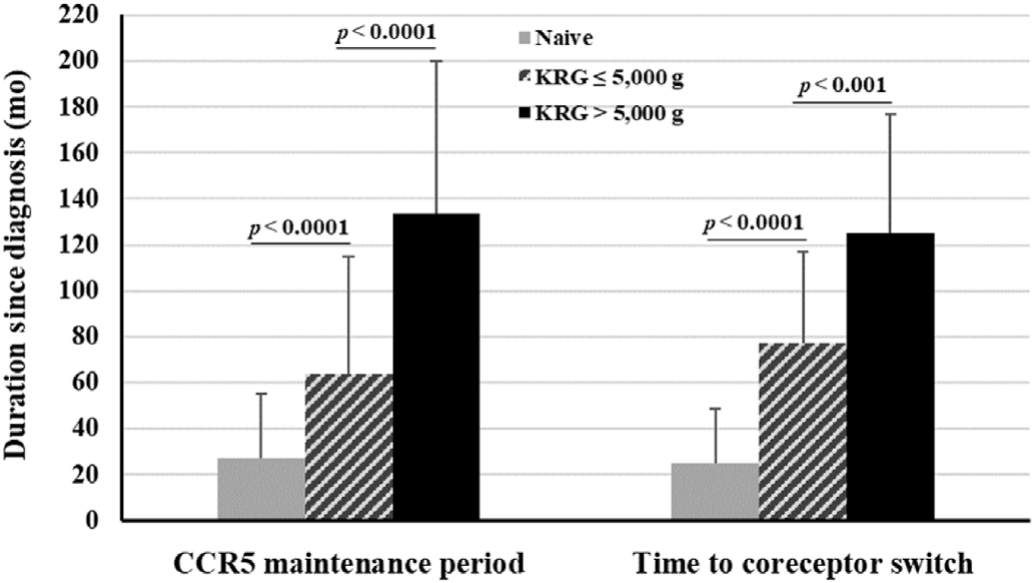
Relationships of CCR5 maintenance period and time to coreceptor switch with total dosages of Korean Red Ginseng (KRG). Patients were divided into those who took ≤5,000 g and those who took >5,000 g KRG. Total dosages of KRG during the R5 maintenance period and at the time of coreceptor switch were 1,891 ± 1,206 g and 1,989 ± 1,275 g, respectively, in patients who took ≤5,000 g KRG, and 10,767 ± 5,866 g and 9,916 ± 3,624 g, respectively, in those who took >5,000 g KRG.

During the entire study period, 88 patients took KRG and 58 did not. The R5 maintenance period and the time to coreceptor switch were 2.98-fold and 3.46-fold longer, respectively, in the former than in the latter (Table 2). Subdivision of the 88 KRG-treated patients into those treated with ≤5,000 g and > 5,000 g KRG showed that the R5 maintenance period was 2.08-fold longer in patients administered > 5,000 g KRG (P < 0.0001). Moreover, the time from diagnosis to coreceptor switch was significantly delayed in patients administered >5,000 g KRG (P < 0.001) (Fig. 3).

### 3.3 Correlation between KRG and coreceptor switching in 20 hemophiliacs infected with the same virus

The correlation between amount of KRG and FPR was higher in the 20 patients with hemophilia (HP; *r* = 0.48, P = 0.059) than in the 58 patients with tropism assessed in three or more samples (*r* = 0.37). Further analysis of the 20 patients with HP showed that the R5 maintenance period was 4.0-fold longer in those treated with KRG than in those naïve to KRG (128 ± 46 versus 32 ± 35 months; P < 0.01), as was the time from diagnosis to coreceptor switch, which was 2.90-fold longer. These 20 patients with HP had been infected with HIV-1 present in contaminated DCF manufactured from plasma obtained from two paid plasma donors. These 20 patients were diagnosed 18 ± 13 months after primary infection [19]. Investigation of the time to coreceptor switching in these 20 patients showed that FPR decreased over time in most, with coreceptor switching occurring in 14 (70%) patients over 10 years and in 17 (85%) patients over 30 years (Fig. S1). In particular, FPRs in eight of these HP patients (numbers 5, 7, 8, 10, 12, 13, 16, and 17) decreased significantly over time (P < 0.05) (Fig. S2), but increased significantly in HP-14 because of treatment with the highest amount of KRG (*r* = 0.73, P = 0.01) (Fig. S3).

Interestingly, HP patient 9, who was administered a low amount of KRG (1,200 g), showed no increase in FPR, whereas HP patient 14, who received a large amount of KRG (40,698 g), showed a significant increase in FRP (Fig. S1 and S3).

### 3.4 Increased FPR after KRG treatment is associated with slow disease progression

Of the 146 patients, 28 were LTSPs, and 118 were progressors, as previously defined [10,11]. A significantly higher proportion of LTSPs (71.4%; 20/28) than of progressors (16.9%, 20/118) maintained R5 for ≥5 years (P < 0.0001).

Of the 20 patients with HP and known primary infections, five were LTSPs and 15 were progressors. R5 was maintained for ≥10 years in four (80%) of the five LTSPs (HP patients 4, 8, 9, and 14; Fig. S1), but in only three (20%) of the 15 progressors (HP patients 2, 11, and 18; P < 0.05). Strict analysis of the R5 maintenance period for >10 years showed that the proportion of LTSPs who maintained R5 was significantly higher than the proportion of progressors (P < 0.0001).

### 3.5 Effect of subtypes of HIV-1 on coreceptor switch

There was no significant difference in tropism between patients infected with B and non-B subtypes, although the degree of correlation in the 24 patients infected with non-B subtypes was higher (*r* = 0.33, P > 0.05) than in the entire patient cohort (*r* = 0.22, P > 0.05).

## 4 Discussion

The present study evaluated HIV-1 coreceptor tropism in the first 153 patients diagnosed in Korea, including 20 patients with HP, over 30 years. The study was designed to determine whether KRG treatment affects coreceptor switching, as well as the proportion of switching in a cohort of patients with hemophilia, all of whom were infected with a single source of HIV-1. Analysis of this cohort over 30 years showed that coreceptor switch occurred in 17 (85%) patients with hemophilia even during complete viral suppression by ART. The KRG dose was significantly higher in patients in whom tropism was determined in two or more samples than in the first sample alone, indicating a significant positive correlation between FPR and amount of KRG.

KRG treatment extended the R5 maintenance period 2.35-fold, resulting in a 2.42-fold extension of the time to coreceptor switching. Moreover, this increase in FPR after KRG treatment was associated with slow disease progression.

Long-term KRG treatment has been shown to slow the depletion of CD4+ T cells [9] and prolong the longevity [11] of HIV-1 infected patients. In agreement with these findings, the proportion of patients in the present study who maintained R5 for ≥10 years was higher in LTSPs than in progressors (P < 0.0001).

The antiviral activity of KRG may be associated with the mechanism by which it delays coreceptor switching. KRG has been shown to inhibit HIV-1 replication, as shown by its reduction of serum p24 antigen levels and plasma RNA copy number [[26], [27], [28]]. Interestingly, KRG therapy also significantly delayed the development of thymidine analog mutations (TAM)-I to zidovudine (unpublished data). The antiviral effect of some ginsenosides is comparable to that of ribavirin [[29], [30], [31]]. In addition, the proportion of patients who were negative on the OraQuick test was significantly higher in patients treated with GCT (24.1%; unpublished data) than with ART (4%) (P < 0.01) [32]. The antiviral effect of KRG may also be due to its potentiation of cytotoxic CD8+ T cell activity, as prolonged treatment with KRG reduced the serum concentration of soluble CD8 antigen [12]. In addition, ginsenoside Re, a component of ginseng, has been found to prolong the survival of human CD4+ T cells through its regulation of autophagy [33]. Furthermore, the anti-inflammatory effects of ginseng can affect innate and adaptive immunity [34,35].

The G2P interpretation system provides reliable discrimination between R5 and X4 sequences when FPR is set between 1% and 20%. Coreceptor tropism can be affected by the FPR setting, although FPR itself is a property of corresponding viruses. Thus, the effects of KRG on FPR level were analyzed. FPR in patients 89-17, 90-05, 90-18, 90-47, and 93-04 was found to decrease gradually and significantly over time. These findings suggest that FPR is a reliable indicator of the impact of KRG, as FPR correlates with KRG intake.

In this study, the mean time to switching was 96 ± 43 months in 29 ART-naïve patients. Among these 29 patients, the correlation between the amount of KRG and time to switching was significant (*r* = 0.46, P < 0.05). KRG treatment was found to significantly delay coreceptor switching, resulting in a longer mean time to switching in the present study than in previous study [7]. Moreover, the proportion of patients who showed an increase in FPR within 3 years was higher in this study (67.4%) than in a previous report (40%) [7].

This study had several limitations. First, KRG treatment was disrupted several times prior to July 2000, including for 4–5 months after the initial 6-month pilot study (972 g) initiated in November 1991 [36]. These early interruptions may be associated with the weak correlation between the amount of KRG and FPR based on the first sample. We therefore evaluated the correlation between the cumulative amount of KRG and FPR. Second, the time of primary HIV-1 infection was not known for most patients except for those with HP. Third, the number of PCR amplicons per sample differed, although we attempted to obtain three or four amplicons per sample and determine tropism for all sequences. The number of PCR amplicons obtained per specimen was found to correlate with the probability of detecting X4 viruses. Thus, the FPRs of 18 samples showed differences ≥50. Finally, although serum samples were generally analyzed at baseline and PBMCs after 2000, FPR was found to be similar in sera and PBMCs [37].

Long-term intake of KRG has been reported to prevent or delay the progression from non-syncytium-inducing to syncytium-inducing viruses based on the 11/25 rule [38]. The correlation between KRG intake and the annual decrease in CD4+ T cells was found to be significant and higher than the correlation between zidovudine and CD4+ T cells in patients followed-up for >60 months. The mechanism by which KRG prolongs CD4+ T cell populations remains unknown, although KRG was reported to have a significant impact on C2/V3 gene variations [23].

In conclusion, this study showed that long-term treatment with KRG slows the depletion of CD4+ T cells and prolongs the time to coreceptor switching in HIV-1 infected patients by increasing FPR. Additional studies are needed to determine whether KRG induces specific changes in the *env* gene, other than nonspecific internal or gross deletions.

## Declaration of competing interest

The authors declare no conflicts of interest.
