# Supplementary figures and images for: Korean Red Ginseng slows coreceptor switch in HIV-1 infected patients
Source: J Ginseng Res. 2022 Jul 6;47(1):117–22. doi: 10.1016/j.jgr.2022.06.003 (PMC9834003; doi:10.1016/j.jgr.2022.06.003)

|  | | **CD4+ T cell (/μL) (●––––●)** |  | 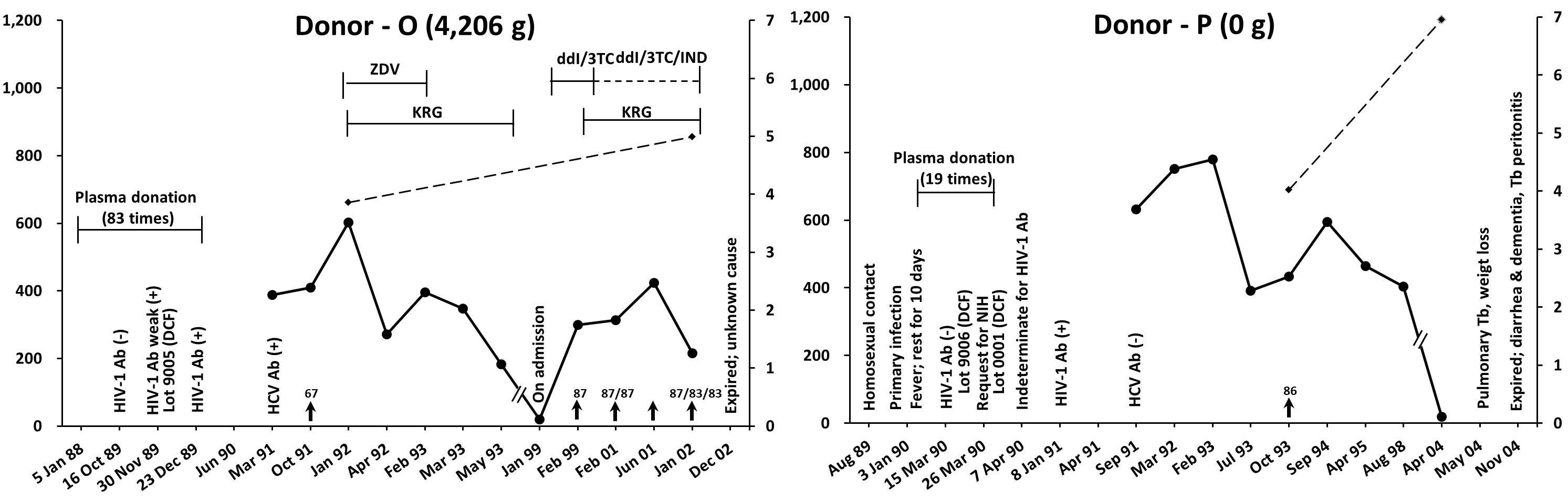  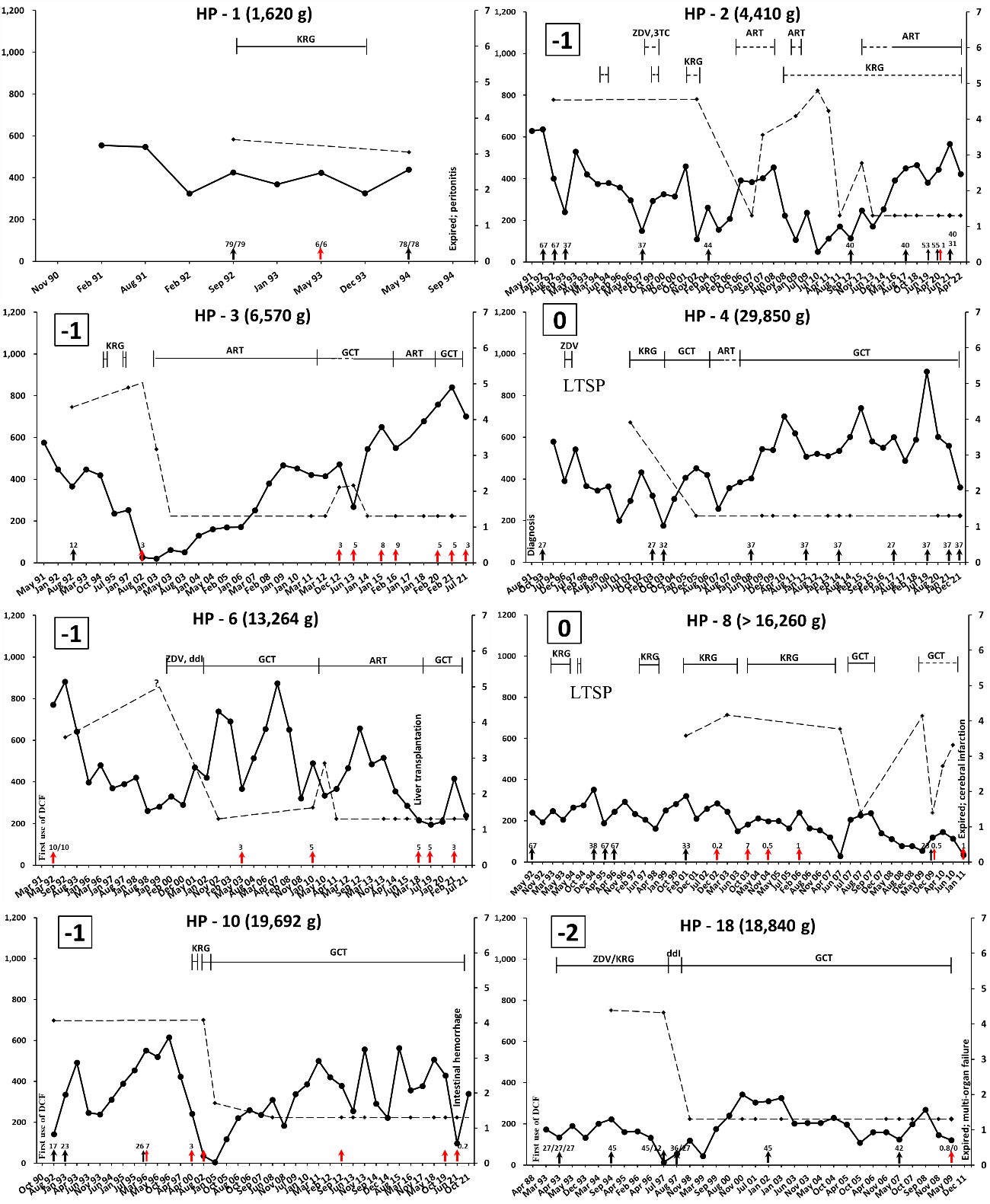 | **RNA copy (log/mL) (◆－－－◆)** | | --- | --- | --- | --- | |  |  | **Sampling date** |  | |  |
| --- | --- | --- | --- | --- | --- | --- | --- | --- | --- | --- |

Fig. S1 (continued)

| **CD4+ T cell (/μL) (●––––●)** |  |  |  | 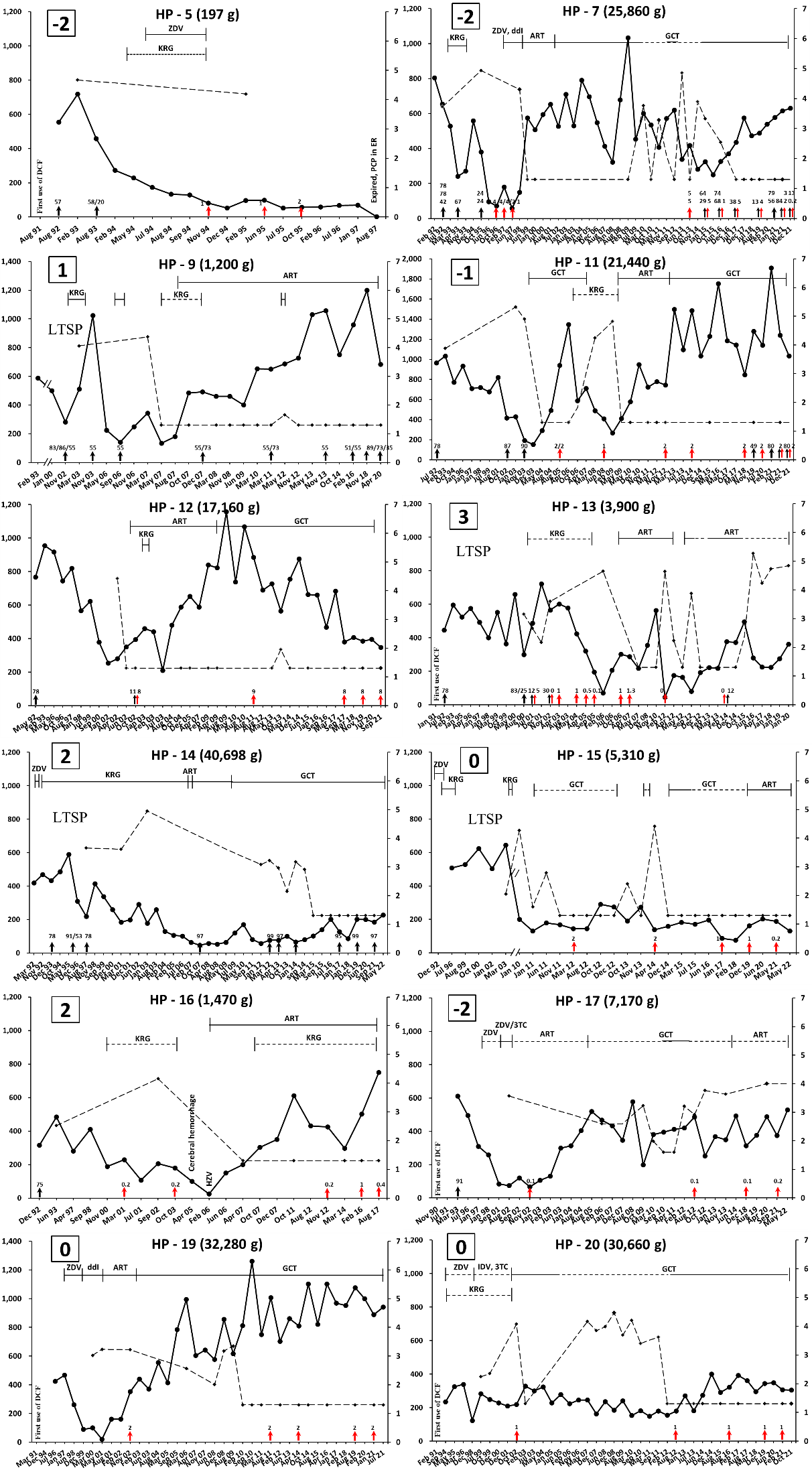 | **RNA copy (log/mL) (◆－－－◆)** |
| --- | --- | --- | --- | --- | --- |
|  |  |  |  | **Sampling date** |  |

Supplement: Multimedia component 1 [file mmc1.docx]

| **CD4+ T cell (/μL) (●––––●)** | 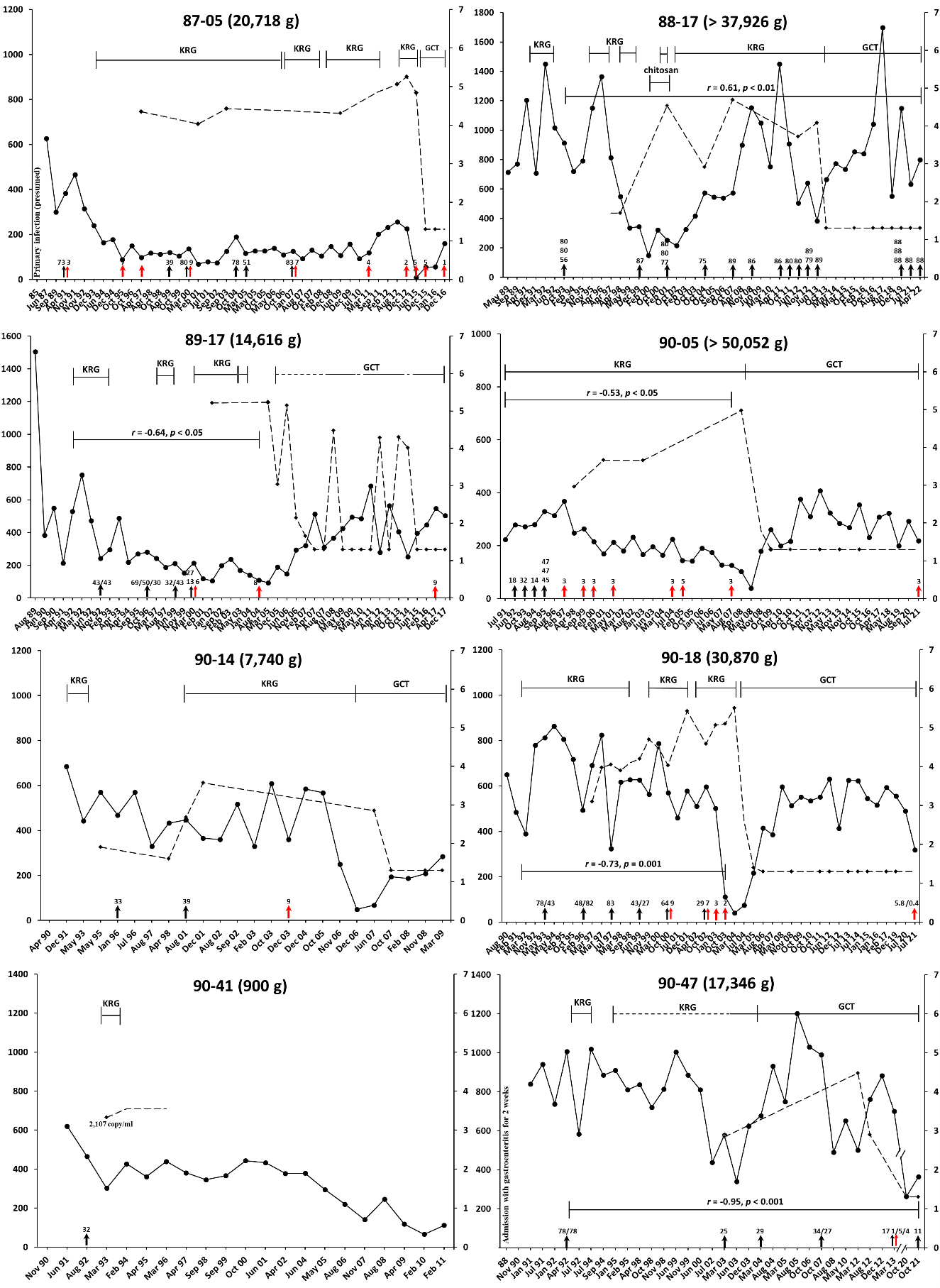 | **RNA copy (log/mL) (◆－－－◆)** |
| --- | --- | --- |
|  | **Sampling date** |  |

Fig. S2 (continued)

| **CD4+ T cell (/μL) (●––––●)** | 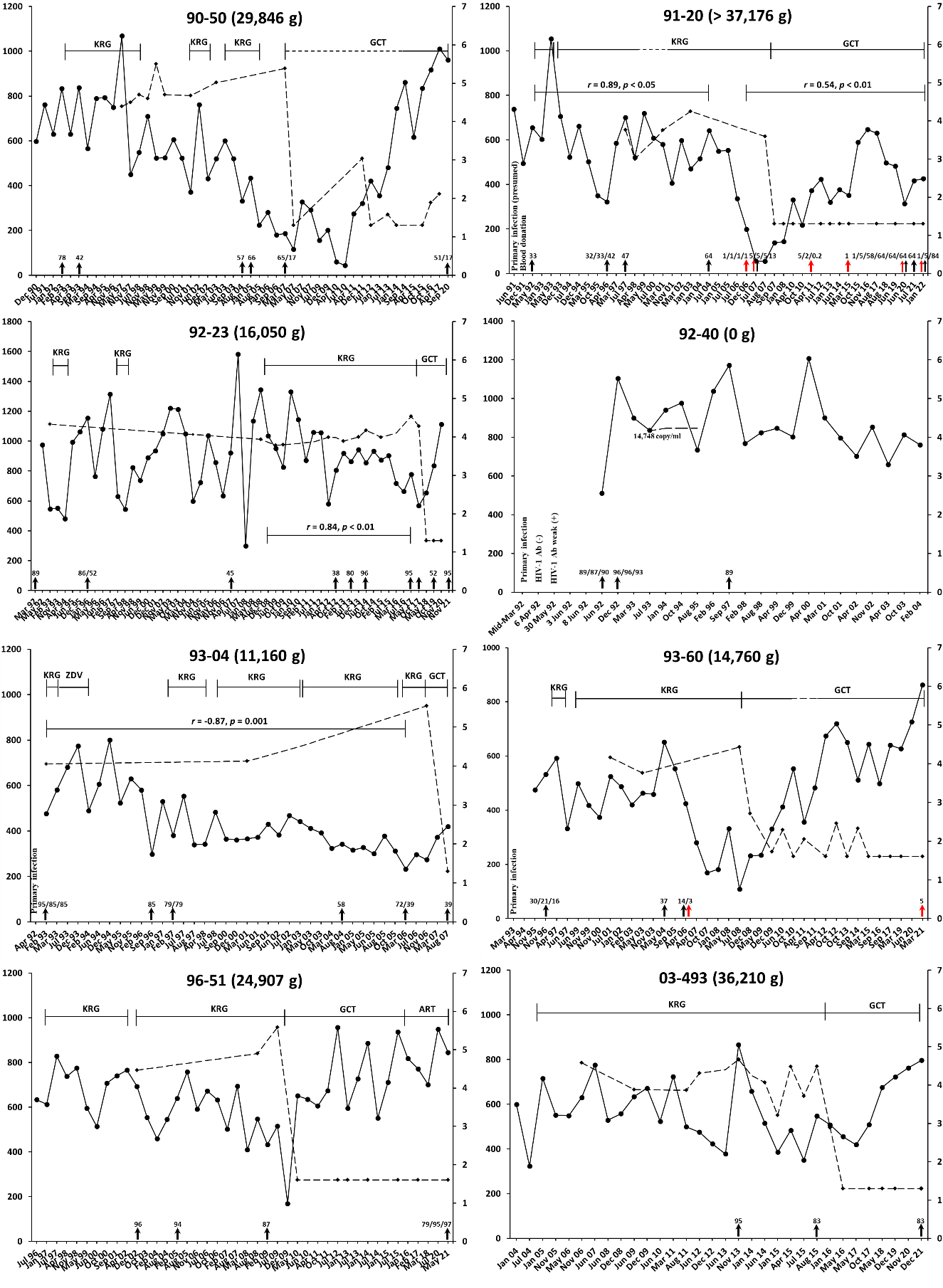 | **RNA copy (log/mL) (◆－－－◆)** |
| --- | --- | --- |
|  | **Sampling date** |  |

Supplement: Multimedia component 2 [file mmc2.docx]

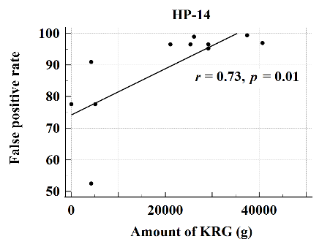

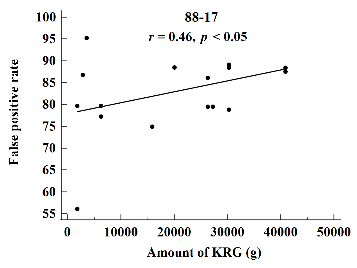

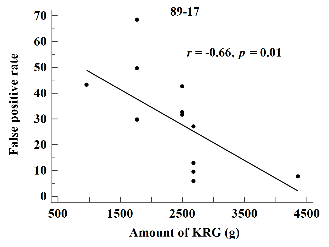

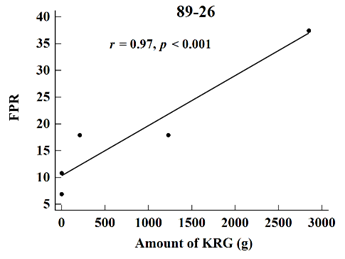

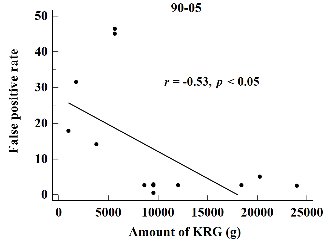

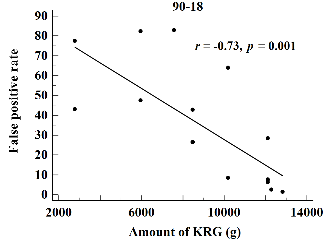

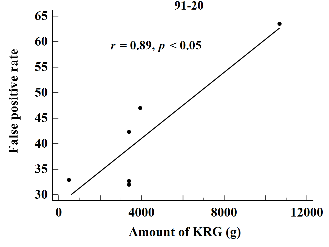

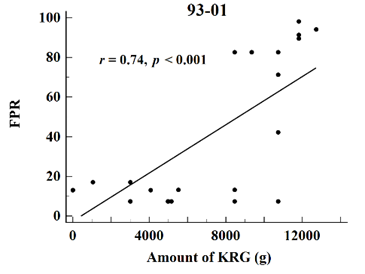

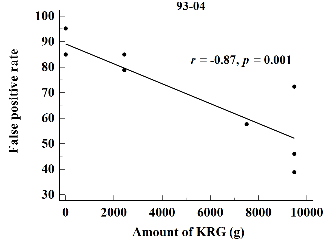

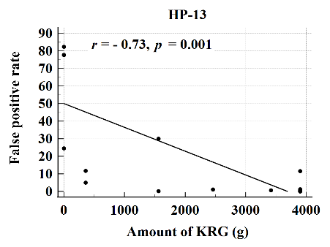


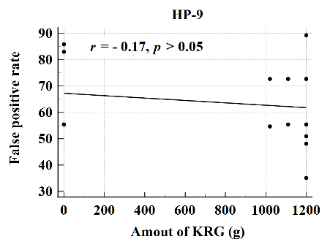


**Fig. S3**

Supplement: Multimedia component 3 [file mmc3.docx]
